# Seizure after IDDS implantation – unrecognized intracranial hypertension associated with intracranial metastases: A case report

**DOI:** 10.1097/MD.0000000000050406

**Published:** 2026-08-28

**Authors:** Yangyang Li, Chunmei Wu, Zehao Liu, Qing Zhong

**Affiliations:** a Department of Anesthesiology, The Affiliated Hospital, Southwest Medical University, Luzhou, Sichuan Province, China; b Department of Anesthesiology, The People’s Hospital of Jianyang, Chengdu, Sichuan Province, China.

**Keywords:** cerebral edema, intracranial hypertension, intracranial metastases, intrathecal drug delivery system, seizure

## Abstract

**Rationale:**

The external intrathecal drug delivery system (IDDS) provides targeted analgesia and serves as an effective therapy for refractory cancer pain. Its known complications include post-dural puncture headache, device malfunction, granuloma formation, and others. Seizures induced by occult intracranial hypertension following IDDS implantation have rarely been reported. This case report describes a cancer-pain patient who developed seizures after IDDS implantation.

**Patient concerns:**

A 66-year-old female with metastatic rectal cancer suffered from refractory lumbosacral and lower-extremity cancer pain and received external IDDS implantation for pain relief. Seizure-like symptoms occurred 19 hours after surgery. Although intracranial metastatic lesions were present preoperatively, routine evaluation revealed no obvious clinical manifestations of intracranial hypertension.

**Diagnoses:**

Secondary epilepsy was considered likely to be induced by marked intracranial-pressure fluctuations associated with jet-like cerebrospinal fluid outflow during intraoperative puncture. Emergency cranial CT revealed multiple intracranial metastatic nodules with peritumoral cerebral edema and scattered intracranial air. Several potential contributing factors, including direct tumor-related epileptogenesis, opioid neurotoxicity, chemotherapy-associated leukoencephalopathy, and acute stroke, were relatively less probable in this case.

**Interventions:**

Intrathecal morphine infusion was suspended immediately. Intravenous diazepam and mannitol were administered to reduce intracranial pressure, phenobarbital sodium and sodium valproate were used to control convulsions. Oral levetiracetam was given for long-term seizure prophylaxis. After seizure control, IDDS therapy was restarted under close monitoring with a reduced basal infusion rate.

**Outcomes:**

The patient regained consciousness after emergency management and had no further seizure episodes. Satisfactory analgesia was achieved with intrathecal therapy. Due to progressive primary malignancy, the patient’s family opted for voluntary discharge on postoperative day 8. The patient died of cardiac arrest 3 days after discharge, with no seizure recurrence during that period.

**Lessons:**

Unrecognized preoperative intracranial hypertension in patients with intracranial metastases significantly increases the risk of severe neurological complications after IDDS implantation. Reliance solely on the classic triad of intracranial hypertension is insufficient for preoperative screening. Pre-operative cranial imaging evaluation is strongly recommended for high-risk patients. Intraoperative cerebrospinal fluid loss should be strictly controlled to prevent abrupt changes in intracranial pressure. Clinicians should raise vigilance toward such life-threatening complications.

## 1. Introduction

The external intrathecal drug delivery system (IDDS) is one of the effective approaches for managing refractory cancer pain.^[1]^ This technique involves placing a catheter into the subarachnoid space to deliver analgesics such as morphine directly into the cerebrospinal fluid (CSF), thereby targeting opioid receptors in the spinal cord and brain to achieve potent analgesia. Compared with oral analgesics, IDDS not only significantly improves the rate of pain control in patients with refractory cancer pain but also reduces overall healthcare costs and systemic drug exposure, lowering the incidence of related adverse reactions.^[2]^ However, as a surgical procedure, IDDS carries a series of potential risks. Common complications include post-dural puncture headache, nausea, vomiting, and urinary retention. Moreover, due to the unique structure of the device and its route of administration, several serious adverse events have been reported, such as device malfunction leading to abrupt cessation of drug delivery and consequent severe opioid withdrawal syndrome, as well as catheter-tip granuloma formation resulting in neurological impairment.^[3]^ This article reports a case of a patient with rectal cancer and intracranial metastases who had no preoperative symptoms of central nervous system involvement but experienced a new-onset seizure after implantation of an IDDS.

## 2. Case presentation

### 2.1. General information

The patient is a 66-year-old female admitted to the oncology department with a diagnosis of “rectal cancer with multiple metastases.” She had previously received 12 cycles of FOLFOX plus cetuximab chemotherapy at an external hospital in June 2023. In March 2025, following assessment indicating disease progression, the regimen was switched to FOLFIRI plus bevacizumab for an additional 3 cycles. Due to poor tolerance, treatment was subsequently adjusted to capecitabine plus bevacizumab for maintenance therapy. In May, a reexamination at an external hospital revealed the development of bone and brain metastases, leading to discontinuation of antitumor therapy and a transition to palliative pain management. In July, due to worsening left lumbosacral and left lower limb pain, the patient was transferred to our hospital for further pain management. Physical examination upon admission revealed widespread tenderness over the left lumbar spine, paravertebral region, and left lower extremity. FABERE test (−), Straight Leg Raise and Braggard tests (−). No pathological reflexes were elicited. During pain episodes, the Numerical Rating Scale (NRS; range, 0–10) score was 9, and the Pittsburgh Sleep Quality Index (range, 0–21) score was 17. Laboratory tests showed an elevated white blood cell count (18.55 × 10^9^/L) with no other significant abnormalities. Thoracolumbar magnetic resonance imaging (MRI): osteophyte formation in the thoracic and lumbar spine; multiple abnormal signals in the thoracic, lumbar, and sacral vertebral bodies and appendages, suggestive of metastases; disc protrusion from L2/3 to L4/5; spinal canal and lateral recess stenosis; and compression of the corresponding dural sac and nerve roots. Following admission, the patient received standard morphine-based analgesic therapy, with the dose of oral morphine hydrochloride sustained-release tablets gradually increased to 150 mg every 12 hours, supplemented by subcutaneous morphine hydrochloride injection (10 mg) when pain control was inadequate (NRS ≥ 4). Nevertheless, analgesia remained suboptimal, and adverse effects such as nausea, vomiting, and constipation emerged. After thorough discussion between the pain specialist, the patient, and her family, it was decided to proceed with implantation of an external IDDS. It is noting that the family recalled the patient had undergone cranial imaging at an external hospital in June 2025 (specific results were not retained), and the findings were described as showing “-no significant change”- compared to imaging performed in May of the same year. Based on this impression of stable disease, the family declined the recommended cranial contrast-enhanced MRI during the current admission.

### 2.2. Surgical procedure

**Figure 1.**
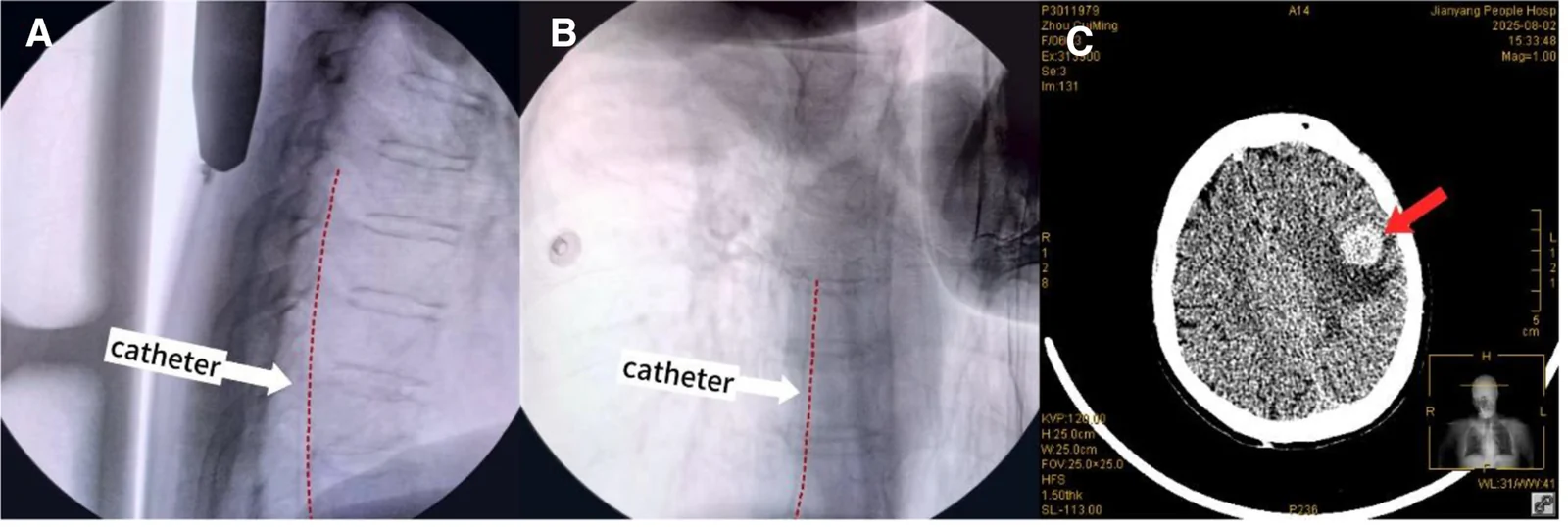
(A, B) Catheter position in anteroposterior and lateral views, with the catheter tip positioned at the T8 level. (C) Post-seizure cranial CT: Multiple slightly hyperdense nodules are observed intracranially. The arrow indicates the largest lesion, measuring approximately 2.0 cm, surrounded by edema with scattered intracranial air present.

The IDDS (model ZS2-I-Q-1.1/0.7–1000, Beijing Yuetong Medical Equipment Co., Ltd.) was implanted under local anesthesia with the patient in the left lateral decubitus position. A 15-gauge Tuohy needle (90 mm in length) was inserted into the L3–L4 intervertebral space. Clear, jet-like CSF flow was observed, prompting the surgical team to expedite subsequent steps to minimize CSF loss. After confirming entry into the subarachnoid space, an intrathecal catheter (outer diameter 1.1 mm, inner diameter 0.7 mm, length 1000 mm) was advanced through the needle lumen under C-arm fluoroscopic guidance, with its tip positioned at the T8 level (Fig. 1A, B). The guidewire (length 1000 mm) and puncture needle were then withdrawn. Free CSF backflow was verified, and contrast agent was injected through the catheter to reconfirm proper tip placement. A subcutaneous tunnel and pump pocket were created. The catheter was threaded through the tunnel to the pocket site, connected to the infusion port, and secured. Following complete hemostasis, a butterfly needle was inserted into the reservoir and connected to an external electronic infusion pump (model REHN-11, Jiangsu Renxian Medical Technology Co., Ltd., China). The procedure was completed uneventfully. The patient maintained stable vital signs throughout, with an estimated blood loss of <5 mL, and reported no significant discomfort.

### 2.3. Postoperative course

The patient was transferred back to the ward in stable condition postoperatively. The initial intrathecal solution consisted of morphine hydrochloride (100 mg) and bupivacaine hydrochloride (112.5 mg) in 0.9% sodium chloride, with a total volume of 260 mL. A continuous basal infusion was started at 0.3 mg/h and gradually titrated upward, which was equivalent to approximately 1/300 of the patient’s total preoperative oral morphine dose. Within 4 hours postoperatively, assessment revealed significant pain relief: the NRS score decreased from 9 to 3, and the Pittsburgh Sleep Quality Index score decreased from 17 to 12. The patient’s mood improved, and she was able to engage in simple communication.

At 19 hours postoperatively, the patient suddenly developed right upper limb twitching, followed by confusion and unresponsiveness. Examination revealed elevated blood pressure (180/100 mm Hg), bradycardia (55 beats/min), and decreased SpO_2_ (88%). Oxygen was immediately administered, and 10 mg diazepam was given intravenously to control convulsions. An emergency blood glucose test result was 8.9 mmol/L, excluding hypoglycemia as a possible cause. Blood gas and electrolyte analysis revealed no significant abnormalities. Neurological examination showed fixed gaze, sluggish pupillary light reflex, trismus, and mildly increased muscle tone in all 4 limbs. Given the patient’s history of advanced metastatic rectal adenocarcinoma, the multidisciplinary expert resuscitation team, which included neurologists, initially considered secondary epilepsy as the likely diagnosis. The intrathecal morphine infusion was immediately suspended, and 250 mL of mannitol was administered intravenously for dehydration and intracranial pressure (ICP) reduction. Approximately 20 minutes later, she gradually regained consciousness and was transferred for emergency cranial CT. While in the CT examination room, the patient experienced another seizure. Immediate intervention included an intramuscular injection of 0.1 g phenobarbital sodium, an intravenous bolus of 10 mg diazepam, and a 1 g infusion of valproate sodium delivered via an intravenous micropump. Emergency cranial CT revealed multiple slightly hyperdense nodules, the largest measuring approximately 2.0 cm in diameter, surrounded by edema and scattered intracranial air (Fig. 1C). About 30 minutes later, the patient recovered consciousness, and an urgent neurological consultation confirmed the diagnosis of secondary epilepsy. Treatment was continued with valproate sodium 1 g via intravenous micropump infusion, supplemented with oral levetiracetam 0.5 g twice daily for seizure management. Subsequently, under close monitoring, IDDS therapy was restarted at a lower basal rate (0.2 mg/h) and gradually titrated upward. No further seizures occurred thereafter, and pain control remained effective (NRS = 3). On postoperative day 8, due to progression of the patient’s underlying disease, the family opted for voluntary discharge. The patient passed away on the third day after discharge due to cardiac arrest, with no recurrence of seizures during this period.

## 3. Discussion

A systematic literature search was conducted across PubMed, Embase, Web of Science, Google Scholar, CNKI, and Wanfang databases. The search strategy employed a combination of key terms: (“intrathecal drug delivery system” OR “IDDS”) AND (“cancer pain”) AND (“epilepsy”), covering all records from database inception to December 2025. Potential eligible studies and references from relevant reviews were manually screened. Inclusion criteria encompassed randomized controlled trials, cohort studies, case series, case reports, and letters published in Chinese or English. Despite the comprehensive search, no clinical studies directly addressing secondary epilepsy following IDDS implantation were identified. This absence may be associated with the following factors: publication bias, where negative results or small-sample studies remain unpublished; Terminology heterogeneity, where “epilepsy” might be subsumed under broader categories such as “rare adverse events” without separate reporting. Given the limitations of the existing evidence, this report aims to explore the potential predisposing factors and underlying pathophysiological mechanisms of post-IDDS seizures in this case, focusing on factors such as ICP shifts, direct tissue stimulation, intracranial morphine effects, chemotherapeutic agents, and stroke.

First, direct stimulation of brain tissue by tumors themselves may induce seizures. Such seizures are often triggered by tumor infiltration, an altered local chemical microenvironment, and abnormal electrical discharges, and their risk of occurrence is closely correlated with tumor type and location.^[4]^ Compared with primary intracranial tumors, intracranial metastases carry a lower seizure incidence, which may be attributed to their frequent multifocal distribution and predilection for the posterior fossa.^[5]^ Notably, seizures typically manifest in patients with brain parenchymal metastases, while leptomeningeal involvement also confers a recognized risk of epileptic episodes.^[6]^ A study enrolling 171 patients with metastatic colorectal cancer identified intracranial metastases in 14.6% of subjects, among whom 76% had asymptomatic intracranial lesions. This phenomenon can be explained by the short interval from initial diagnosis to intracranial dissemination and the small size of metastatic lesions,^[7]^ which also accounts for why some patients lack prominent central nervous system manifestations before obvious intracranial hypertension or seizure onset. Tumors continuously irritate brain tissue and induce epilepsy with a chronic onset. A long-standing epileptogenic microenvironment persists around the lesion, and seizure frequency gradually increases with disease progression, accompanied by recurrent episodes.^[8]^ However, the clinical course of this patient was markedly inconsistent with such characteristics. First, the patient had no seizure history prior to IDDS implantation, lacking the pathological basis of recurrent epileptiform discharges induced by prolonged tumor stimulation. Second, acute seizure occurred only 19 hours after surgery; symptoms temporarily relieved after symptomatic management but relapsed shortly afterwards. Nevertheless, no further seizures recurred until the patient’s death, which contradicts the typical pattern of tumor-related epilepsy where seizure frequency rises over time. Therefore, tumor stimulation was excluded as the underlying cause of the patient’s convulsions.

Second, previous reports have documented that high-dose intravenous or intrathecal morphine can induce seizures, which may be attributed to the toxic effects of sulfite preservatives contained in morphine formulations.^[9]^ Another potential mechanism involves normorphine, an intermediate metabolite of morphine. This substance accumulates in patients with renal failure and provokes myoclonus,^[10]^ representing a major risk factor for seizure induction in patients with advanced cancer, particularly those with acute renal dysfunction secondary to renal metastases. Studies have demonstrated that low-concentration morphine exerts anticonvulsant effects, whereas high-dose morphine selectively activates μ and κ opioid receptor subtypes to produce neuroexcitatory effects and trigger seizures.^[11]^ The morphine dosage and IDDS parameters administered to the patient were all within the normal reference range.^[12]^ Accordingly, we consider it unlikely that the seizure episode was induced by IDDS-related opioids.

Third, patients receiving systemic chemotherapy may also develop seizures. Central nervous system adverse reactions induced by chemotherapeutic agents are relatively rare, yet case reports have demonstrated that platinum-based drugs can trigger demyelination of central nervous cells to precipitate seizures, or induce hypomagnesemia, hyponatremia, and other electrolyte disorders that lower the seizure threshold.^[13]^ Administration of 5-fluorouracil may lead to leukoencephalopathy, characterized by confusion, coma, and seizures.^[14]^ Capecitabine adopted in the chemotherapy regimen of this patient can be hydrolyzed into 5-fluorouracil in both tumor cells and normal hepatocytes,^[15]^ and capecitabine-associated leukoencephalopathy may occur during any chemotherapy cycle,^[16]^ consequently provoking seizures. However, no such lesions were observed on the patient’s cranial imaging, so this etiology was excluded.

Forth, seizures can also occur secondary to stroke. Cancer patients, especially newly diagnosed individuals, face an elevated risk of both hemorrhagic and ischemic stroke, with ischemic stroke more prevalent among patients with neurological tumors.^[17]^ Direct tumor effects, coagulation disorders, infections, as well as diagnostic and therapeutic interventions for malignancy are all contributing factors to stroke in cancer populations.^[18]^ No obvious hemorrhagic or ischemic infarct lesions were detected on cranial CT of this patient; therefore, stroke is excluded as an inducing factor for her seizures.

**Figure 2.**
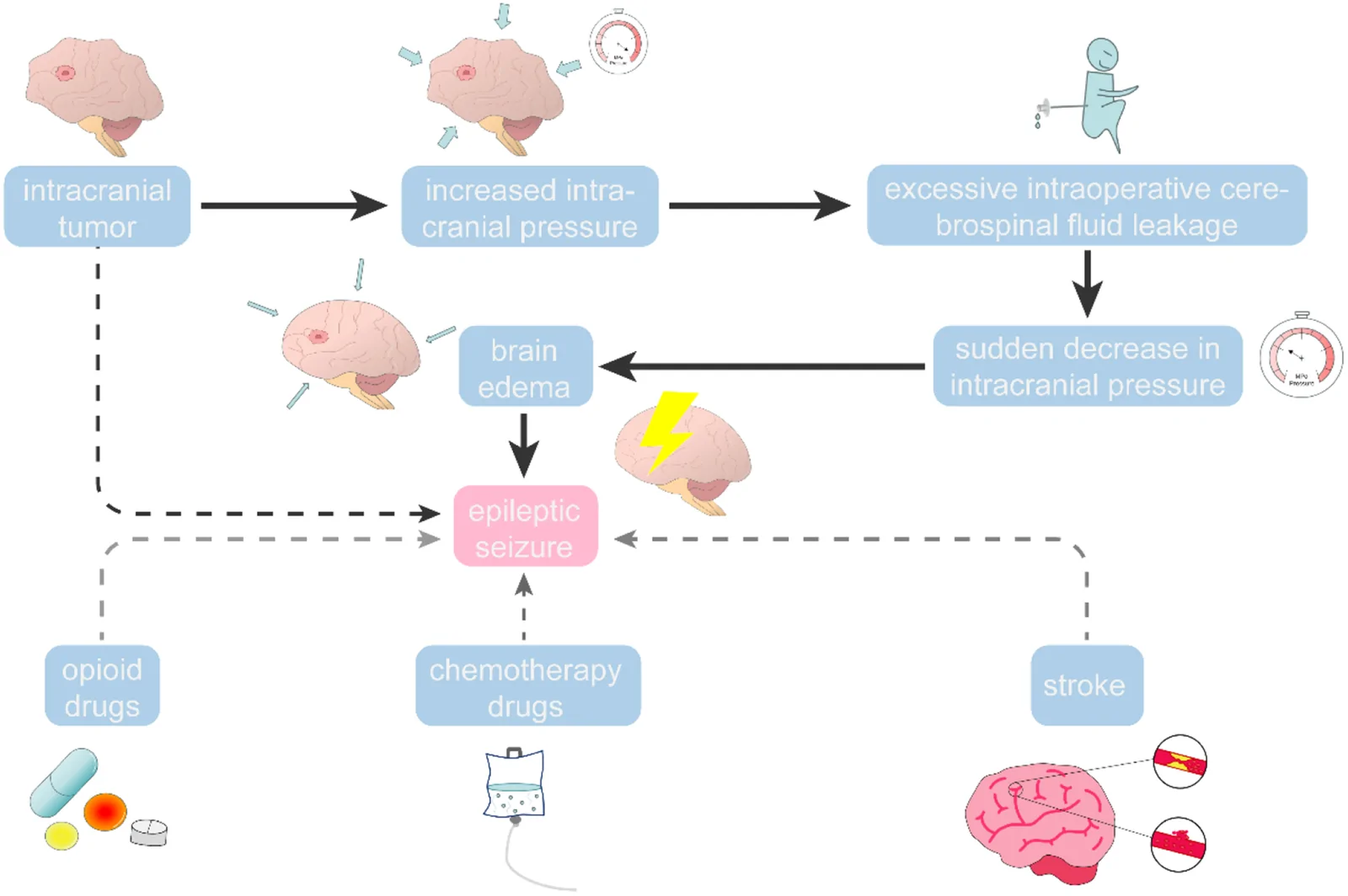
Workflow and etiological analysis of postoperative seizure.

Finally, rapid shifts in ICP may trigger seizures. ICP is determined by brain tissue, CSF, and cerebral blood volume.^[18]^ Within a certain range, these 3 components interact to maintain relatively stable ICP. When the volume of 1 component rises, such as in cerebral edema, the volumes of the other 2 components, especially CSF, will decrease to keep ICP constant.^[19]^ Once this compensatory capacity is exhausted, ICP will rise or fall.^[20]^ Patients with intracranial metastases may have concomitant intracranial hypertension, which mainly arises from the following factors: as intracranial tumors grow, tumor cells gradually displace adjacent healthy tissues. Combined with tumor-induced vascular abnormalities, the blood-brain barrier is disrupted, leading to massive plasma leakage into tumor tissue and resultant cerebral edema.^[21]^ Both the mass effect of tumors and cerebral edema contribute to elevated ICP.^[22]^ Tumor compression can obstruct CSF circulation, impairing normal absorption and reflux of produced CSF and resulting in obstructive hydrocephalus, which further elevates ICP.^[23]^ Compression or occlusion of cerebral blood vessels may also lead to increased ICP.^[24]^ Classic intracranial hypertension manifests as the triad of headache, vomiting, and papilledema, and disease progression may be accompanied by confusion, convulsions, and Cushing’s response.^[25]^ However, the patient in this case presented none of the above central nervous system symptoms preoperatively. The only objective high-risk clue was multiple intracranial metastatic lesions confirmed on preoperative imaging. Previous literature has indicated that CSF pressure can directly reflect ICP levels.^[26]^ Jet-like outflow of CSF was observed during intraoperative lumbar puncture, which directly indicated occult intracranial hypertension already existed before surgery. The patient had long relied on CSF compensation to maintain a relatively stable hypertensive equilibrium. Rapid massive CSF loss during lumbar puncture directly disrupted this steady state, triggering 2 types of pathological injuries. First, abrupt ICP reduction markedly increased the transvascular pressure gradient of cerebral vessels. Fluid extravasated into the brain parenchyma along the pressure gradient and aggravated focal edema, causing asymmetric cerebral shift, which may progress to cerebral herniation in severe cases.^[27]^ Second, rapid CSF loss led to post-puncture intracranial hypotension,^[28]^ which further exacerbated vasogenic cerebral edema, reduced cerebral perfusion, and induced hypoxia and energy metabolic disorders, ultimately triggering postoperative seizures.^[29]^ Previous studies have reported seizures secondary to intracranial hypotension after spinal surgery in patients with normal preoperative ICP.^[30]^ In contrast, occult preoperative intracranial hypertension in our patient caused sharp ICP reduction, which served as the predominant trigger for seizures. Emergency cranial CT obtained after seizure onset only revealed unilateral focal edema without bilateral diffuse cerebral parenchymal involvement, and the imaging manifestations were inconsistent with posterior reversible encephalopathy syndrome.^[31]^ This further demonstrates that seizures in this case were most likely mediated by drastic ICP fluctuations (Fig. 2).

This case report suggests that early identification of intracranial hypertension is required for patients with cancer pain who are scheduled for IDDS implantation and carry high-risk factors for brain metastases. Intracranial hypertension cannot be screened merely by the classic triad of headache, vomiting, and papilledema.^[25]^ Completion of the latest cranial MRI before surgery serves as an effective evaluation method, as midline shift and hydrocephalus formation all indicate an increased risk of intracranial hypertension.^[32]^ If intracranial hypertension secondary to intracranial metastatic tumors is detected and assessed at an early stage, ICP can be reduced by adjusting body temperature and ventilation parameters or administering hypertonic saline.^[19]^ A study by Pedersen et al found that ICP measured in the lateral decubitus position was higher than that in the supine position.^[33]^ However, a report by Abel et al noted no statistically significant difference in CSF pressure between the prone position, which is commonly adopted during surgery, and the lateral decubitus position.^[34]^ Therefore, positional adjustment during IDDS implantation may offer limited benefits for such patients. In addition, puncture manipulation should be slowed intraoperatively and the outflow rate of CSF strictly controlled to avoid drastic ICP fluctuations caused by instantaneous massive CSF loss. Although a comprehensive analysis has largely ruled out direct stimulation by intracranial tumors, excessive intrathecal morphine, chemotherapy-related encephalopathy, and acute stroke as potential triggers for postoperative seizures in this patient, we still need to establish a standardized, rapid differential diagnosis pathway and management protocol in clinical practice. Seizures induced by intracranial tumor infiltration into brain tissue usually have an insidious onset with progressively worsening symptoms, and the primary therapeutic options include surgical resection of lesions or local radiotherapy.^[8]^ Morphine administered within standardized therapeutic doses rarely triggers seizures.^[35]^ If drug-related seizures are highly suspected, clinical symptoms usually relieve rapidly after discontinuation of the suspected agent.^[36]^ When using special medications with high epileptogenic risks, clinicians must adhere to a gradual dose titration regimen starting at a low initial dose.^[37]^ Seizures associated with chemotherapeutic agent-induced central nervous system injury are often accompanied by diffuse abnormal signals in cerebral white matter.^[13]^ To date, no specific therapeutic regimen exists for chemotherapy-related leukoencephalopathy; the core intervention is discontinuation of the offending chemotherapeutic drug, and combined administration of glucocorticoids and antioxidants can ameliorate clinical manifestations and imaging lesions.^[38]^ Acute stroke can be rapidly classified via cranial imaging. Once confirmed, thrombolysis, interventional therapy, or surgical intervention should be initiated immediately to halt irreversible cerebral tissue injury to the greatest extent.^[39]^ This hierarchical differential diagnostic framework facilitates rapid identification of the exact etiology of post-IDDS seizures and enables targeted clinical management.

## 4. Conclusion

For terminal cancer pain patients undergoing IDDS implantation, vigilance against ICP elevation secondary to intracranial metastases might be essential. Preoperative cranial imaging should be completed, and if elevated ICP is identified, appropriate pressure-reducing measures should be implemented. This helps prevent pathological changes during puncture that could adversely affect patient outcomes and enhances perioperative safety. Notably, epileptic seizures secondary to intracranial lesions are triggered by multiple complex factors. The temporal distribution characteristics of seizures alone cannot accurately identify the underlying etiology. Comprehensive clinical evaluation incorporating cranial imaging, ICP monitoring, and other indicators is therefore required.

## Author contributions

**Visualization:** Zehao Liu.

**Writing – original draft:** Yangyang Li, Chunmei Wu.

**Writing – review & editing:** Qing Zhong.
